# Organic Liquids-Responsive β-Cyclodextrin-Functionalized Graphene-Based Fluorescence Probe: Label-Free Selective Detection of Tetrahydrofuran

**DOI:** 10.3390/molecules19067459

**Published:** 2014-06-06

**Authors:** Huawen Hu, John H. Xin, Hong Hu, Xiaowen Wang, Xinkun Lu

**Affiliations:** The Hong Kong Polytechnic University, Hung Hom, Kowloon, Hong Kong, China; E-Mails: hua.w.hu@connect.polyu.hk (H.H.); wang.xiaowen@polyu.edu.hk (X.W.); xk.lu@polyu.edu.hk (X.L.)

**Keywords:** β-cyclodextrin, graphene oxide, chemical reduction and functionalization, functionalized graphene, Rhodamine B, fluorescence quenching, fluorescence switch-on, fluorescence probe, sensor, sensing platform

## Abstract

In this study, a label-free graphene-based fluorescence probe used for detection of volatile organic liquids was fabricated by a simple, efficient and low-cost method. To fabricate the probe, a bio-based β-cyclodextrin (β-CD) was firstly grafted on reduced graphene surfaces effectively and uniformly, as evidenced by various characterization techniques such as Ultraviolet/Visible spectroscopy, Fourier transform infrared spectroscopy, X-ray diffraction, thermogravimetric analysis, scanning electron microscopy and transmission electron microscopy. The subsequent inclusion of Rhodamine B (RhB) into the inner cavities of the β-CD grafted on the graphene surfaces was achieved easily by a solution mixing method, which yielded the graphene-based fluorescent switch-on probe. In addition, the gradual and controllable quenching of RhB by Fluorescence Resonance Energy Transfer from RhB to graphene during the process of stepwise accommodation of the RhB molecules into the β-CD-functionalized graphene was investigated in depth. A wide range of organic solvents was examined using the as-fabricated fluorescence probe, which revealed the highest sensitivity to tetrahydrofuran with the detection limit of about 1.7 μg/mL. Some insight into the mechanism of the different responsive behaviors of the fluorescence sensor to the examined targets was also described.

## 1. Introduction

Graphene, a monolayer of carbon atoms patterned into a hexagonal π-conjugated honeycomb structure, has attracted a great deal of attention across various scientific disciplines. This is owing to its unique physical and chemical properties [1], such as its giant specific surface area [2], ultra-high electrical conductivity [3], thermal conductivity [4] and mechanical strength [5], superior thermal and chemical stabilities [6,7], and remarkable fluorescence quenching property [8,9], among others [10]. Great efforts have thus been dedicated to the applications of graphene using these properties, among which optical fluorescence-based detection applications have recently received intense interest based on graphene oxide (GO) and graphene platforms because of the easy fabrication, functionalization and sensitization of the platforms [11]. A large number of GO- and graphene-based sensing platforms, particularly GO-based ones, have been fabricated through various kinds of strategies based on the fluorescence quenching properties of GO [12,13,14], chemically reduced graphene [15], and other kinds of graphene derivatives [16]. A range of target analytes have been explored using these sensing platforms, with the interests mainly focused on the selective assay of metal ions, such as Mn^2+^ [11], Hg^2+^ [17,18], Pb^2+^ [19] and K^+^ [20,21], and biomolecules such as enzymes [15], RNA [12,22], DNA [13,14,23,24], concanavalin A [9], cysteine [25], biotin [26], thrombin [27], matrix metalloproteinase 2 [28] and cholesterol [29]. Despite the progress achieved, graphene-based fluorescence sensors or probes used for detection of volatile organic liquids can rarely be found. Moreover, most GO- and graphene-based sensors are fabricated by dye labeling techniques, with expensive single-stranded DNA (ssDNA) or aptamers as the probe, which thereby causes the preparation process to be complicated and costly. Additionally, the ssDNA and aptamers are prone to denaturation, thus leading to instability of the as-fabricated probes [11]. The wide use of GO as the basic platform can also make the GO-based probes unstable due to the presence of the labile oxygen-containing functional groups on the GO surface [30]. On the other hand, lower fluorescence quenching efficiency is provided by GO as compared to that of graphene [8,9], indicative of a lower signal-to-background ratio and thus a lower fluorescence sensitivity of the GO-based sensor [14]. Consequently, the exploration of alternative label-free graphene (instead of GO) -based sensing platforms at a low cost, and with high chemical and thermal stabilities and fluorescence sensitivity, is highly desirable.

The widespread use of GO as the basic platform is due to its high yielding synthesis (using naturally abundant graphite as the starting material) and its stable colloid nature in aqueous solution favored by hydrophilicity and electrostatic repulsions of the abundant oxygen functionalities [31]. This water solubility and stability can play an important role in the successful construction of complex structures for subsequent sensing applications [15]. In addition, the strong interactions between the functional groups of a fluorescence probe and the oxygen functionalities on GO surfaces can allow the close contact between the fluorophores and the nanoquencher GO and hence facilitate the fluorescence quenching of the probe. This can be explained by the fact that the fluorescence intensity has been demonstrated to be distance-dependent, and it can be enhanced by a factor of 7.5 by increasing the distance between the fluorescence probe and graphene surface from 4 to 7 nm [32]. 

To maintain the main advantages of GO in the fabrication of a graphene-based fluorescence probes, such as low cost, high yield and water solubility, it is expected that the graphene will be prepared from GO by a reduction/deoxygenation process. However, removal of the oxygen functional groups from GO surfaces via various reduction approaches to prepare reduced graphene results in an irreversible aggregation and agglomeration, and even restacking to the bulk graphite structure, which severely limits the applications of the thus-reduced graphene [33]. This can be attributed to the decreased hydrophilicity and the strong interplanar van der Waals forces and π-π stacking interactions [34]. To prepare effective graphene dispersions comparable to GO colloids, much attention has been paid to graphene surface modifications, either non-covalent [35,36] or covalent [37,38,39], in addition to other feasible strategies of reducing GO under given conditions [31,40]. For instance, our previous work has demonstrated effective aqueous dispersions of polydopamine-functionalized graphene [37] and low-temperature thermally functionalized graphene [40,41], as well as satisfactory organic dispersions of organically-modified graphene derivatives [40]. The well-synthesized graphene dispersion not only has a higher fluorescence quenching efficiency as compared to the GO colloid, but also affords an enhanced π-π stacking interaction with commonly-used aromatic probes due to the restored π-conjugated structure of graphene after reduction of GO.

In this work, a bio-based β-cyclodextrin (β-CD) is utilized as the covalent modifier to fabricate a functionalized graphene with high water solubility by a one-pot functionalization and reduction process. The β-CD-modified and functionalized graphene will hereafter be called CD-G. The as-prepared CD-G can be used directly as the sensing platform that can quench the fluorescence dye efficiently by inclusion of the dye molecules into the CD-G and hence provide close contact of the dye with the graphene moiety, a super nanoquencher. By selectively driving the dye molecules out of the CD-G cavities based on host-guest competition interactions with given target molecules, a sensing probe can be constructed using CD-G as the basic platform. In the present work, Rhodamine B (RhB) was adopted as the fluorescence reporting molecule for CD-G to form an inclusion complex of CD-G and RhB, *i.e.*, the resulting fluorescence probe or denoted as CD-G-R. A wide spectrum of organic liquids, including water-soluble and -insoluble organic solvents, were subsequently employed as the target molecules to attack CD-G-R in order to examine the sensitivity and selectivity of CD-G-R to these molecules. To the best of our knowledge, this is the first report of the use of a graphene-based sensing platform for detection of the small organic molecules in liquid form, even though a large body of work has been dedicated to the detection of various metal ions and biomolecules using GO- or graphene-based sensing systems, as mentioned before. The present strategy for fabrication of the sensing platform CD-G-R is very simple, efficient, and cost-effective. Moreover, high fluorescence turn-on sensitivity and fair selectivity to tetrahydrofuran (THF) can be observed. THF is a polar ether and is a widely used solvent for many applications such as processing solvent, polymer production, and component in stripping fluids, cleaning fluids, functional fluids, coatings, lacquers and corrosion inhibitors [42]. A notable amount of THF entering the environment can cause contamination and health problems, and even explosions by considering that it is readily soluble in water and has a relatively low boiling point [43]. Acute toxicity [44] and carcinogenicity [45] have been found for THF. Activities of enzymes [43,46] and anaerobic microbes [47] have also been validated to be inhibited by THF, which implies degraded biodegradability of wastewaters containing THF. These findings therefore verify the considerable importance of the present low-cost sensing platform capable of detecting THF, which holds great potential for solvent leakage detection of tanks and piping systems. Using fluorescence spectroscopy, the quantitative analysis of organic solvent concentration in water can also be realized with our developed graphene-based sensor. The present work on a simple, efficient, and cost-effective fabrication of a graphene-based fluorescence probe will also pave the way for the development of other label-free graphene-based sensing platforms for various sensing, monitoring and detection applications.

## 2. Results and Discussion

### 2.1. Main Content of This Study

**Figure 1:**
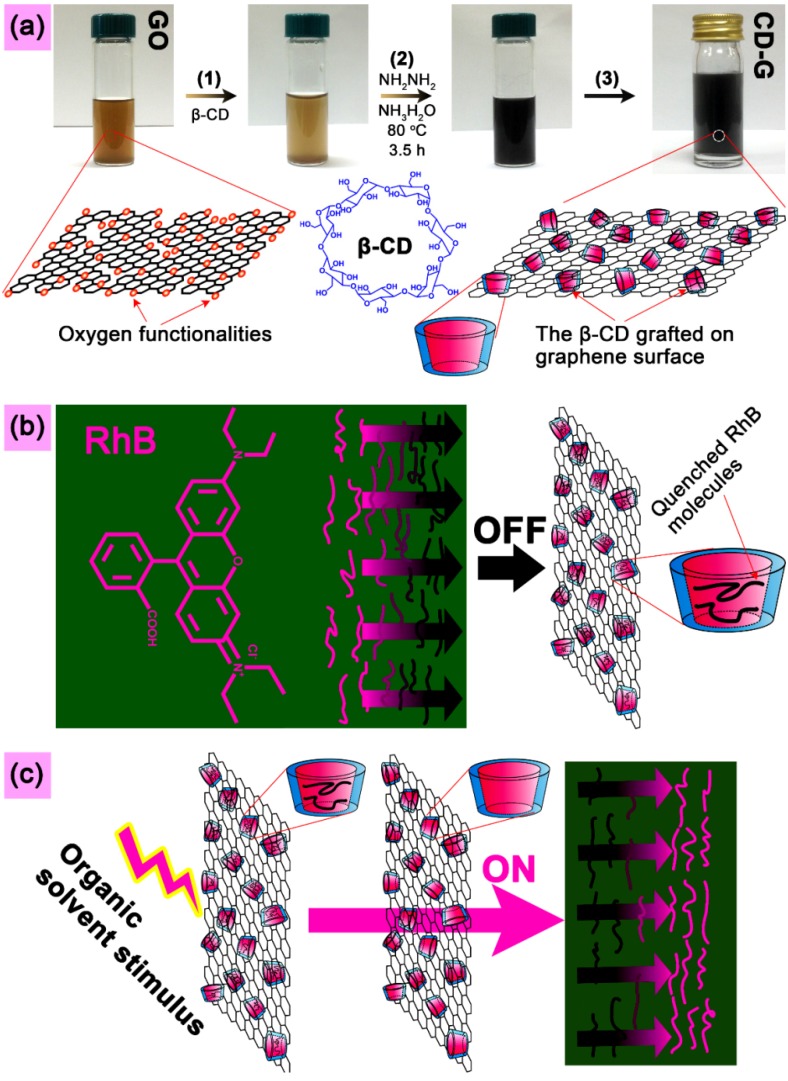
(**a**) Digital images and schematic illustrations showing the primary experimental procedure for fabrication of CD-G, as well as the structural models of GO, β-CD and CD-G. (**b**) Schematic diagram used to explain the quenching of the RhB fluorescence by CD-G in molecular scale, as well as showing the complex structure of CD-G and RhB. (**c**) Schematic presentation of the fluorescence switch-on process, along with molecular scale details.

The photo images and schematic illustrations given in Figure 1 show the main content of the present study, including synthesis of GO and CD-G, fluorescence quenching of the RhB solution with the as-prepared CD-G water dispersion to produce the inclusion complex of CD-G and RhB, namely CD-G-R, and fluorescence switch-on detection of a range of organic solvents using CD-G-R. Figure 1a presents the synthesis process of the CD-G water dispersion. The homogeneous water dispersion of GO with yellow-brown color was synthesized by a modified Hummers method, followed by ultrasonication in water. The β-CD solution and ammonia were mixed into the GO dispersion, and the one-pot reduction and functionalization of GO were then conducted with hydrazine as the reducing agent, leading to a stable black dispersion. After dialysis, filtration, washing and re-dispersing treatment, the black aqueous dispersion of CD-G at a concentration of 0.265 mg/mL was obtained and used as the subsequent quenching agent for RhB solution. Figure 1b depicts schematically the quenching of RhB fluorescence by CD-G. The aromatic RhB can be readily accommodated into CD-G, resulting in the close contact between the RhB molecules and the super-nanoquencher graphene, and thus effective quenching of the RhB fluorescence by Fluorescence Resonance Energy Transfer (FRET) from the RhB molecules to the graphene. The FRET mechanism has been widely adopted for explaining the quenching behavior between the pair of energy donor and acceptor [48,49,50,51]. With the quenched inclusion complex of CD-G and RhB, the subsequent fluorescence turn-on detection of various organic liquids can be carried out easily by solution mixing method. Different host-guest competition interactions between these organic solvents and RhB lead to different responsive behaviors and fluorescence switch-on intensities at given excitation and emission wavelengths. The fluorescence turn-on mechanism is schematically shown in Figure 1c. When the RhB molecules are driven out of the cavities, the distance to the graphene surfaces is largely increased, enabling the enhancement of the distance-dependent fluorescence [32].

### 2.2. Structure Analysis of GO and CD-G

**Figure 2:**
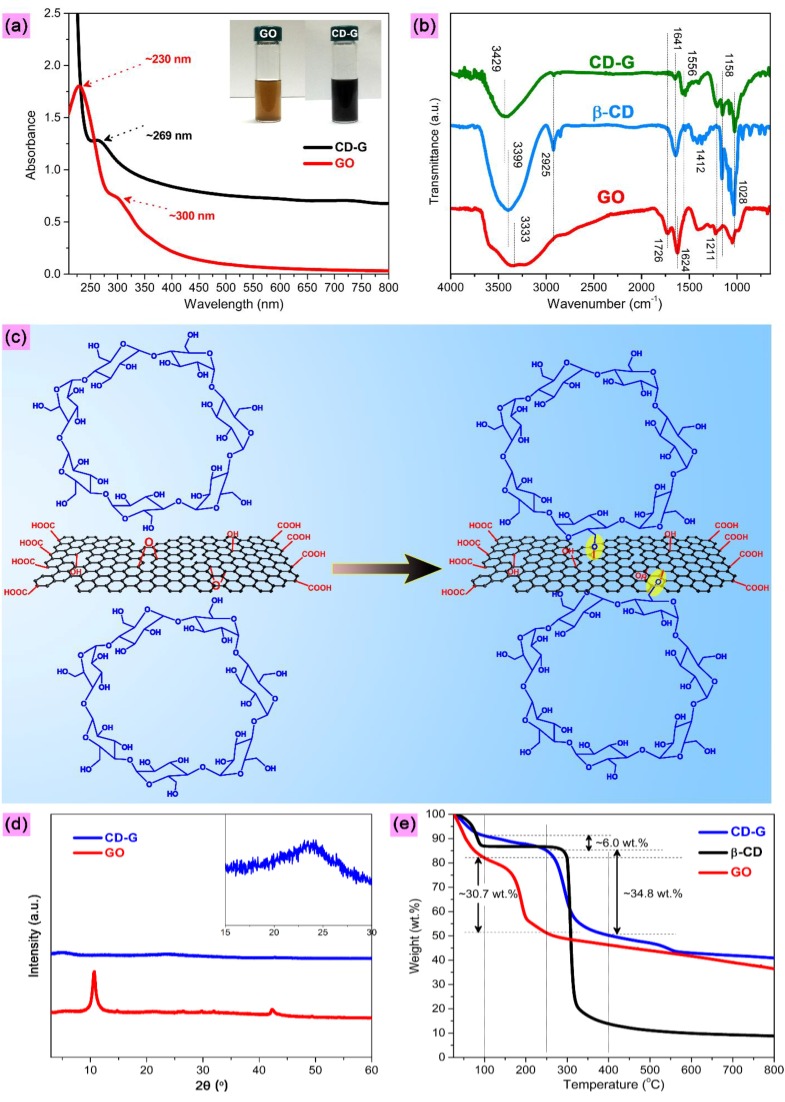
(**a**) UV/Vis spectra of GO and CD-G (inset presents the photo images of the water dispersions of GO before and after chemical reduction and functionalization with hydrazine and β-CD). (**b**) FTIR spectra of GO, β-CD and CD-G. (**c**) Proposed mechanism of chemical grafting of β-CD onto the graphene surfaces through the reaction of hydroxyl groups present in the exterior walls of β-CD with the epoxy groups existing on the GO surfaces (the generated chemical bonds between β-CD and graphene are highlighted with yellow shading). (**d**) XRD patterns of GO and CD-G (inset shows the magnified XRD pattern of CD-G). (**e**) TGA curves of GO, β-CD and CD-G.

The structure characterization results for GO and CD-G are shown in Figure 2. There are two typical absorption peaks in the Ultraviolet/Visible (UV/Vis) spectrum of GO, as shown in Figure 2a. The one centered at approximately 230 nm corresponds to the π→π* transitions of aromatic C-C bonds, and the other shoulder peak located at about 300 nm is attributed to the n→π* transitions of C=O bonds [52]. The peak at 230 nm is red-shifted to about 269 nm after the chemical reduction and functionalization of GO, which indicates the restoration of the electronic conjugation within the CD-G sheets [52]. The inset clearly shows the color change from yellowish-brown for GO to black for CD-G, which is an indication of the effective reduction of GO to the reduced graphene [53]. Figure 2b depicts the Fourier transform infrared (FTIR) spectra. As for GO, typical absorption bands can be observed at approximately 3,333 (O–H), 1,726 (C=O in COOH), 1,624 (bending vibration, from water), and 1,000–1,410 cm^−1^ (C–O in C–OH/C–O–C) [54]. This indicates that the abundant oxygen-containing functional groups are present on GO surfaces. In the FTIR spectrum of the pure β-CD, the absorptions at about 3,399, 2,925, 1,641 and 1,028 cm^−1^ can be indexed to O–H, C–H, O–H (bending vibration, from water), and C–O–C groups, respectively [55]. 

After chemical reduction with hydrazine in the presence of β-CD, the absorptions assigned to carboxyl group, disappears completely and a new absorption band arises at approximately 1,556 cm^−1^ (which can be attributed to the enhanced benzene ring vibrations of CD-G), suggesting the effective deoxygenation and restoration of conjugation structure of graphene. In addition, the absorption peak stemming from water becomes much smaller and blue-shifts to higher wavenumber (from 1,624 cm^−1^ for GO to 1,641 cm^−1^ for CD-G that is the same location as that for β-CD). This is likely due to the high coverage of β-CD on the graphene surfaces, thus changing the water adsorption behavior of graphene to that of β-CD. Because of the disturbance effect of the graphene moiety on the β-CD grafted on CD-G planes, this absorption peak turns to be smaller as compared to that for pure β-CD. It is worth mentioning that the absorption corresponding to O–H group is blue-shifted from about 3,333 (for GO) and 3,399 cm^−1^ (for β-CD) to 3,429 cm^−1^ for CD-G, probably caused by the intermolecular interactions between GO and β-CD such as hydrogen bonding [56]. Moreover, we note that the strong FTIR absorptions resulting from β-CD are the main feature found in the FTIR spectrum of CD-G even after the extensive dialysis and washing treatment of CD-G, which thereby implies that β-CD is not physically adsorbed but chemically grafted on the CD-G surfaces. Figure 2c presents the proposed mechanism of the covalent attachment of β-CD to the graphene surfaces through a ring-opening reaction of epoxide present on GO surfaces with -OH groups existing in the exterior walls of β-CD [29,57]. The X-ray diffraction (XRD) patterns are shown in Figure 2d. Concerning GO, one diffraction peak can be seen at 2θ ≈ 10.7° which can be assigned to GO (001) crystallographic plane (corresponding to an interlayer *d*_001_/spacing of 0.83 nm, being much larger than that of graphite (0.34 nm) due to the incorporation of abundant oxygen functionalities) [58]. It is noted that almost no diffraction peaks can be found for CD-G when compared with that for GO, with only a broad peak centered at 2θ ≈ 24° observable under magnification, which is an indication of the disordered stacking of the reduced graphene sheets [59]. This also suggests that the β-CD has been well incorporated on the graphene surfaces, which can thereby strongly inhibit the regular stacking and aggregation of the reduced graphene layers. Figure 2e provides the thermogravimetric analysis (TGA) curves. As for GO, the first stage of a sharp decrease in weight upon heating occurs with increasing temperature to 100 °C, which can be attributed to the adsorbed water in GO powder because of its highly hydrophilic nature. The drastic decomposition of oxygen functionalities can be found around 200 °C. The pure β-CD also presents a marked weight loss in the temperature range below 100 °C, due to its hydrophilicity derived from hydrophilic groups on the outside wall. A sharp weight loss can be observed around 300 °C for the pure β-CD, which can be due to the decomposition of β-CD. The reduction and functionalization of GO affords a much higher heat resistance to CD-G (as compared with GO). Assuming that the thermal decomposition of oxygen functionalities on the GO surfaces occurred in the temperature range of 100–250 °C, the weight percentage of oxygen functional groups on the GO surfaces can be calculated as approximately 30.7 wt. %, much higher than that on the CD-G surfaces (approximately 6 wt. %). This is an indication of effective deoxygenation of GO after the one-pot reduction and functionalization process. Moreover, the content of the β-CD grafted on CD-G surfaces can be calculated as 34.8 wt. % based on the weight-loss stage assigned to the thermal decomposition of the grafted β-CD. It is worth pointing out that the heat resistance of the β-CD is degraded after grafted onto the CD-G surfaces, with the initial decomposition temperature of about 270 °C calculated by the tangent method [60]. This is likely because the graphene moiety heavily disturbs the intermolecular interactions among the β-CD molecules such as intermolecular hydrogen bonding interactions that plays an important role in stiffening the β-CD molecules.

**Figure 3:**
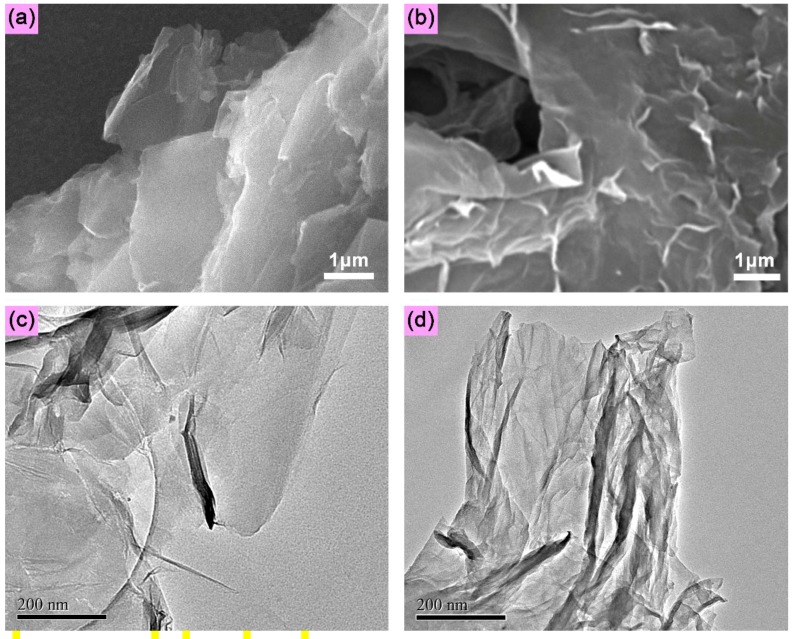
SEM images of GO (**a**) and CD-G (**b**); TEM images of GO (**c**) and CD-G (**d**).

The surface morphologies of GO and CD-G are observed by scanning electron microscopy (SEM), with the images shown in Figure 3a,b, respectively. As compared to that of GO, the SEM image of CD-G presents a more uneven and rough surface topology, as well as a lower transparence, which can be attributed to the β-CD molecules incorporating on the CD-G surfaces. Similarly, the transmission electron microscopy (TEM) image of GO shows a more smooth texture, with higher transparence and much less uneven topology observable on its surfaces (Figure 3c), as compared to that of CD-G (Figure 3d), which is also an indication of the successful grafting of β-CD crystals on the graphene surfaces as for CD-G. Note that both SEM and TEM images for CD-G display a uniform surface morphology and topology. These results thereby imply that the β-CD molecules have been grafted on the graphene surfaces effectively and uniformly, which guarantees the subsequent satisfactory fluorescence sensing applications.

### 2.3. Fluorescence Quenching of RhB with CD-G

**Figure 4:**
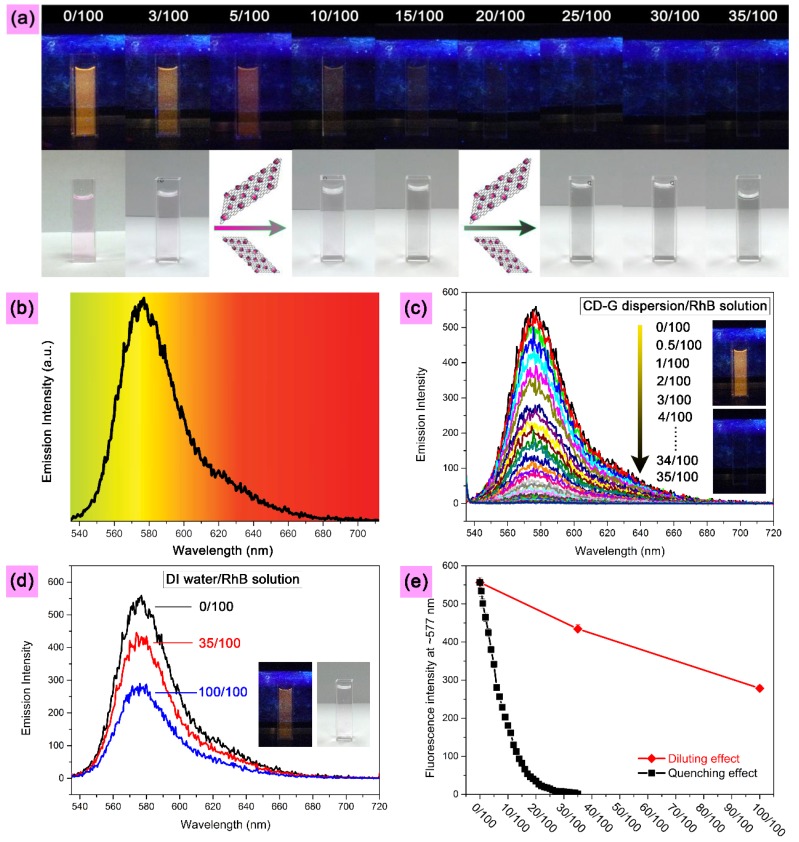
(**a**) Digital images showing gradual fluorescence quenching of the RhB solution by stepwise addition of the CD-G dispersion. Digital images on the top and bottom rows were captured under UV light at 365 nm and daylight, respectively. (**b**) Fluorescence spectrum of the RhB solution, highlighted with the corresponding colors across the wavelengths of 535 to 712 nm (**c**) Dependence of fluorescence emission spectra (excitation wavelength of 530 nm, emission peak centered at approximately 577 nm) on the mixing ratio (*v*/*v*) of CD-G dispersion (0.265 mg/mL) to RhB solution (50 nM). (**d**) To exclude the diluting effect of the DI water moiety of the CD-G dispersion on the quenching efficiency, 35 mL of CD-G dispersion, by which the fluorescence of the RhB solution (100 mL, 50 nM) can be quenched completely, was replaced by 35 mL of DI water, and even higher amount of DI water (100 mL) was also examined. (**e**) Plots of fluorescence intensity at about 577 nm for the mixing system with different volume ratios of RhB solution to CD-G dispersion (error bars were obtained from five parallel experiments). The labels shown in the (**a**) and (**c**) indicate the mixing ratio of CD-G dispersion to RhB solution, e.g., 0/100 and 35/100 denote the neat RhB solution, and the mixture of CD-G dispersion (35 mL) and RhB solution (100 mL), respectively. Similarly, the labels shown in (**d**) represent the mixing ratio of DI water to RhB solution, e.g., 35/100 denotes the mixture of DI water (35 mL) and RhB solution (100 mL).

The CD-G is used firstly as the nanoquencher to quench the fluorescence of the RhB solution and yield the inclusion complex of CD-G and RhB. The results are presented in Figure 4. The digital images taken under UV light at 365 nm and daylight show the gradual quenching of RhB solution, as displayed in Figure 4a. The pristine RhB solution (50 nM) exhibits a light magenta color under daylight, while its color changes to saffron yellow under UV light, which is in line with the fluorescent spectrum with the emissions across the wavelengths of 535 to 712 nm (centered at approximately 577 nm), as shown in Figure 4b. With addition of CD-G dispersions (0.265 mg/mL) to the RhB solution, the light magenta color seen under daylight is decreased stepwise, with a concomitantly gradual increase of gray color. The fluorescent saffron color also disappears gradually with increasing CD-G concentration. The fluorescence almost vanishes when the mixing ratio of CD-G dispersion to RhB solution reaches 15/100 (*v*/*v*). To completely quench the RhB fluorescence monitored by fluorescence spectroscopy, the mixing ratio was further increased to 35/100.

Figure 4c presents the fluorescence spectra to track the quenching process, with the inset showing the digital images captured at the starting and ending points of the process. Considering that the component water of the CD-G dispersion might affect the RhB fluorescence, pure deionized (DI) water was used as control to replace CD-G dispersion. The result shows that the component water has very limited effect on the RhB fluorescence. After even increasing the ratio of DI water to RhB solution to 100/100, there still exists sufficient fluorescence visible by the naked eye (Figure 4d). The comparison plot of fluorescence intensity at about 577 nm against the mixing ratio between the systems of CD-G and RhB and DI water and RhB is also given in Figure 4e. As a result, the efficient quenching of RhB by CD-G is well demonstrated. More importantly, the RhB fluorescence can be quenched controllably based on FRET from RhB to graphene through changing the mixing ratio of CD-G to RhB. In addition, note that the initial increase of the content of the CD-G dispersion can linearly decrease the fluorescence of RhB solution, followed by gradual decrease of the quenching rate.

### 2.4. Fluorescence Sensing Test on the Organic Liquids

**Figure 5:**
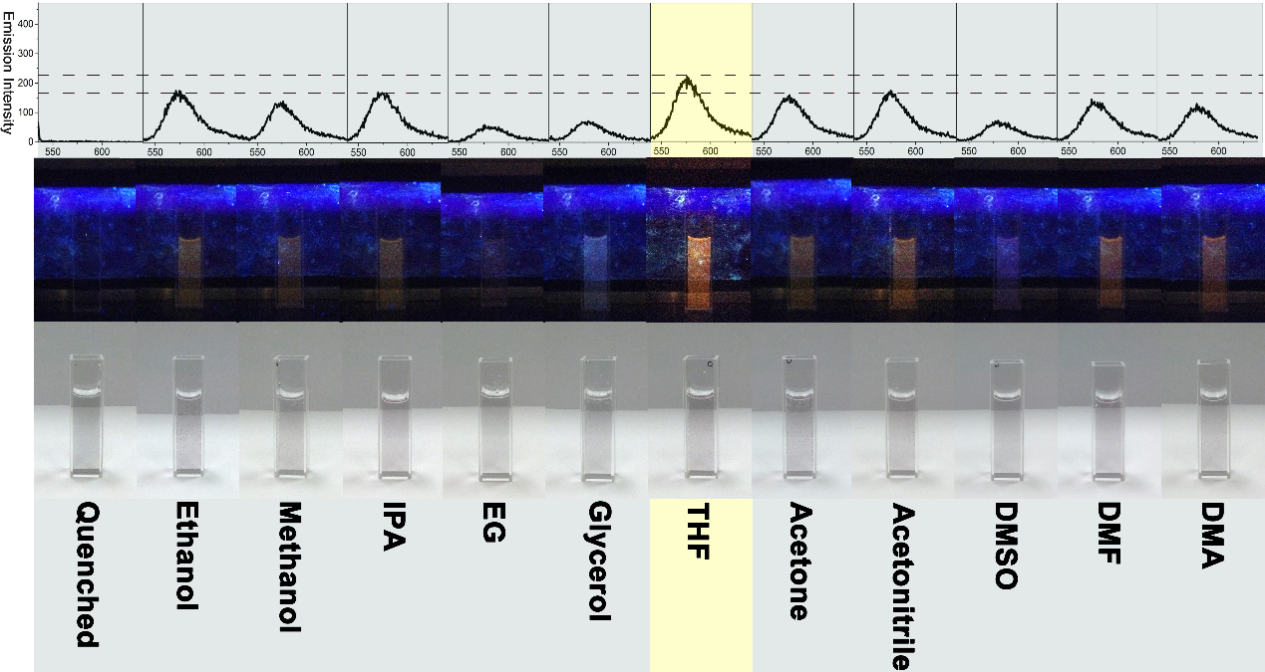
The fluorescent spectra (excitation wavelength of 530 nm, emission peak centered at about 577 nm) are shown in the top row. The corresponding photo images captured under UV light at 365 nm and day light are presented in the middle and bottom rows, respectively. The volume ratio of quenched RhB solution to organic solvent is kept at 2/1. The system with the solvent THF is indicated by yellow shading. The full names of Quenched, IPA, EG, THF, DMSO, DMA and DMA are given as follows: Quenched: quenched RhB solution with CD-G dispersion; IPA: isopropanol; EG: ethylene glycol; THF: tetrahydrofuran; DMSO: dimethylsulfoxide; DMF: dimethyl formamide; DMA: dimethyl acetamide.

Using the completely quenched inclusion complex of CD-G and RhB as the starting sensing platform, the subsequent detection of different organic liquids are performed. A range of water-soluble organic solvents was firstly detected, with the results shown in Figure 5. 

**Figure 6:**
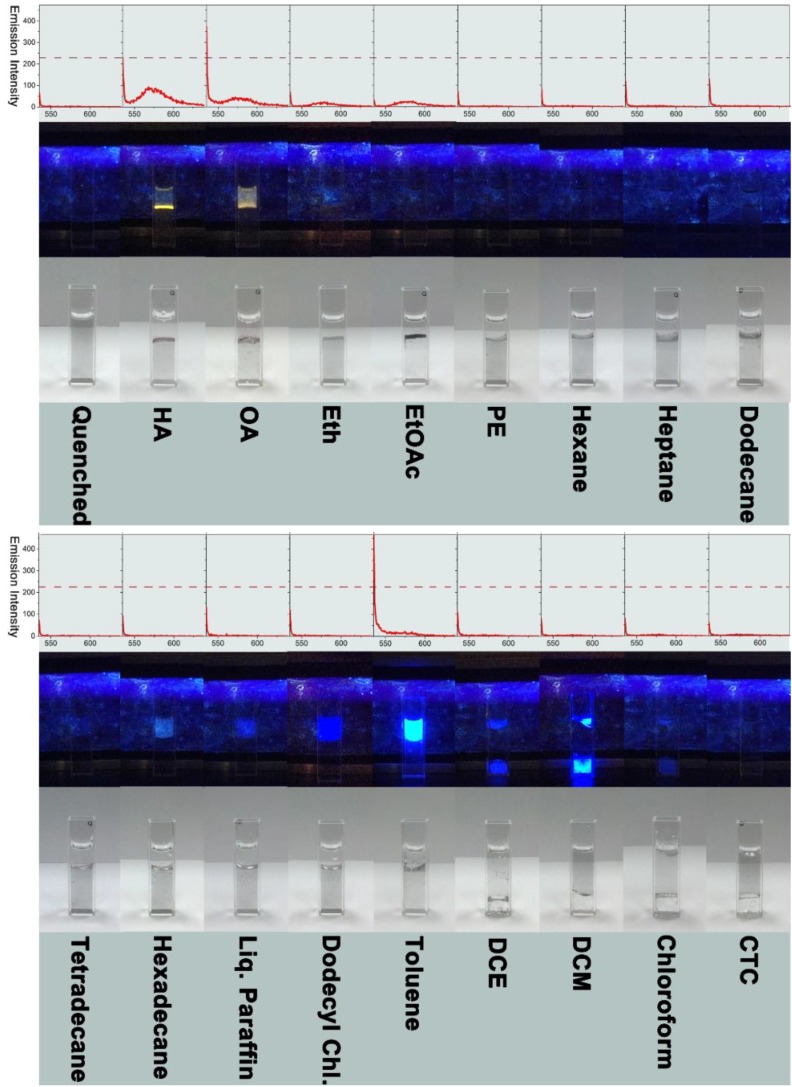
The fluorescence spectra (excitation wavelength of 530 nm, emission peak centered at approximately 577 nm) are shown in the top row. The corresponding photo images captured under UV light at 365 nm and day light are presented in the middle and bottom rows, respectively. The full names of HA, OA, Eth, EtOAc, PE, Liq. Paraffin, Dodecyl Chl., DCE, DCM and CTC are given as follows: HA: hexyl alcohol; OA: 1-octanol; Eth: diethyl ether; EtOAc: ethyl acetate; PE: petroleum ether; Liq. Paraffin: liquid paraffin; Dodecyl Chl.: dodecyl chloride; DCE: dichloroethane; DCM: dichloromethane; CTC: carbon tetrachloride.

**Figure 7:**
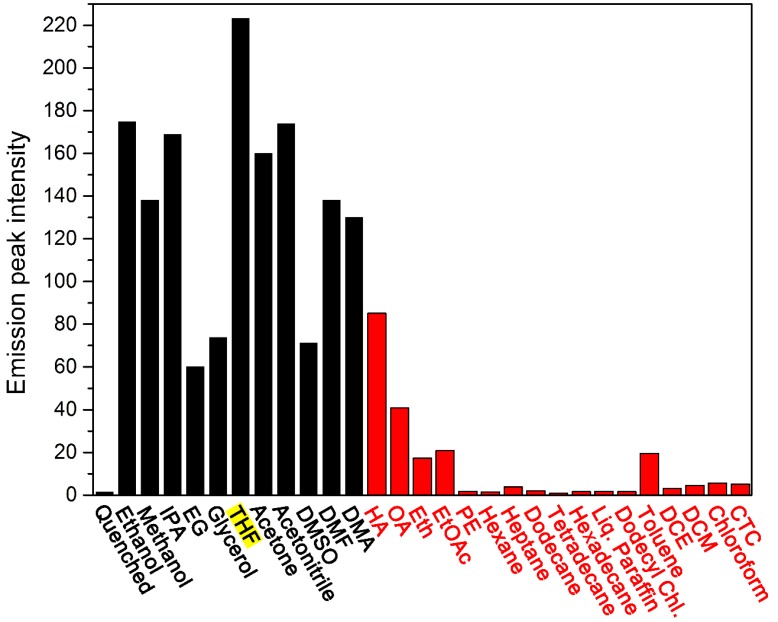
Comparison histogram of typical fluorescence emission intensity against the systems with different organic liquids. The black and red bars represent the systems with water–soluble and –insoluble organic liquids, respectively.

The C1-C3 monohydric alcohols, that is methanol, ethanol, and isopropanol, can turn on the fluorescence to a decent extent observable by the naked eye (C2 and C3 monohydric alcohols perform a little better than C1 monohydric alcohol in switch-on efficiency). The C2 dihydric (ethylene glycol) and C3 trihydric (glycerol) alcohols show a lower fluorescence switch-on efficiency as compared to the C1-C3 monohydric alcohols. This can be attributed to the fact that the increased hydroxyl groups impart higher polarity to the ethylene glycol and glycerol, which reversely reduce the competition with RhB to accommodate into CD-G because of its higher affinity to the hydrophilic outside wall (with abundant hydroxyl groups) than to the hydrophobic inner cavity of β-CD. Acetone and acetonitrile have a comparable turn-on efficiency relative to C2 and C3 monohydric alcohols, in this case ethanol and isopropanol, respectively. In addition, DMF and DMA have a similar turn-on capacity for the quenched RhB to the C1 monohydric alcohol, namely methanol (DMF shows a little higher capacity than DMA according to the fluorescence spectra). DMSO exhibits a parallel turn-on effect as compared with C2 dihydric and C3 trihydric alcohols. Moreover, THF shows an overwhelming performance over all the other examined water-soluble organic solvents, as well as over all the examined water-insoluble organic solvents (see the following Figure 6 and Figure 7). 

The intensity of the switch-on fluorescence at about 577 nm by THF is around 220, which is much larger than that by the second-best solvent, in this case ethanol (around 174). The best match with the molecular structure of inner cavity can be used to explain for the best performance of THF among the examined organic liquids since the inner surface structure of β-CD lined with the cyclic ether-like anomeric oxygen atoms and the C3-H and C5-H hydrogen atoms (see Figure 1) is close to the cyclic ether structure of THF.

Figure 6 provides the fluorescence switch-on results for the targets of the water-insoluble organic liquids. As can be seen, except for dichloroethane, dichloromethane, chloroform and carbon tetrachloride (organic layer formed at the bottom due to their higher relative density than aqueous phase), all the other examined water-insoluble solvents form the upper organic layer because of their lower relative density. Note that C6 (hexyl alcohol) and C8 (1-octanol) monohydric alcohols show the best switch-on efficiency among all the investigated water-insoluble solvents, followed by ethyl acetate, toluene and diethyl ether, based on the switch-on fluorescence spectra. The C6 alcohol performs better than C8 alcohol, which might be due to the polarity of C6 alcohol closer to that of the inner cavity structure of β-CD. It is worth mentioning that alkanes, including hexane, heptane, dodecane, tetradecane, hexadecane, petroleum ether (basically the mixture of pentane and hexane), and liquid paraffin (mainly composed of C16-C20 n-alkanes), show very limited switch-on impact, with the fluorescence emission spectra close to that for the starting quenched counterpart. This implies that there exist almost no interactions between these alkanes and β-CD (neither outside walls nor inside cavities). The interactions between the RhB molecules and the alkanes also cannot be found, which can otherwise lead to some fluorescent RhB molecules extracted to the organic layer, leading to the generation of fluorescence to a certain extent. In addition, the chlorinated hydrocarbons show very low effect on the fluorescence behavior. Except for dodecyl chloride (with a close structure to dodecane) being present in the upper layer, all the other examined chlorinated hydrocarbons form bottom organic layer. It is worth pointing out that a bright luminescent upper layer with a light blue color can be observed for the target toluene, which might be corresponding to the fluorescence emission band located at lower wavelength, as shown in the spectrum with a sharp emission intensity at the wavelength of 535 nm or less.

To summarize the fluorescence switch-on effect of all the organic solvent investigated in the present study, a comparison histogram of the emission intensity at about 577 nm is plotted against the mixing system with different organic liquids, with the result presented in Figure 7. The water-soluble solvents have an overall higher fluorescence switch-on efficiency as compared with the water-insoluble solvents, and THF affords the most sensitive fluorescence response.

**Figure 8:**
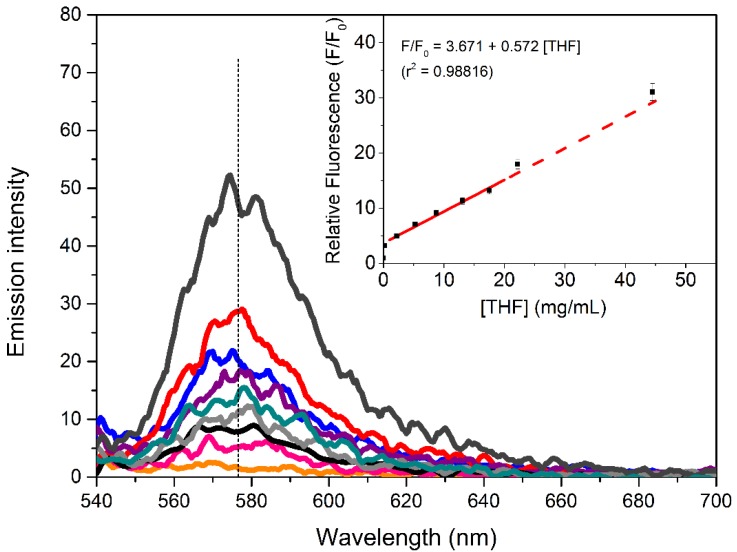
Fluorescent spectra monitoring the fluorescence switch-on process with THF in a range of concentrations. Inset gives the calibration curve for THF detection, where F_0_ and F are the fluorescence intensity in the absence and the presence of THF, respectively.

Considering that the graphene-based sensing platform CD-G-R has a selectivity for THF among the organic liquids examined in the present study at the given excitation wavelength and scanning range. The subsequent calculation of LOD is carried out by monitoring the fluorescence spectra of CD-G-R system upon the addition of increasing concentration of THF (0–44.5 mg/mL), with the result shown in Figure 8. 

The calibration curve of the relative fluorescence (F/F_0_) for detection of THF is given as the inset of Figure 8, where F_0_ and F are the fluorescence intensity in the absence and presence of THF, respectively. Error bars in the calibration curve were obtained from five parallel measurements. The pristine CD-G-R shows nearly no emission in the detected range, probably indicating that all the RhB molecules have accommodated in CD-G and thus lead to a close contact with graphene moiety and highly effective quenching of the fluorescence by FRET from RhB to the graphene. The CD-G-R showed a good linear response of the relative fluorescence intensity with respect to the THF concentration in the range of 0.218–17.5 mg/mL (*r*^2^ = 0.98816) as shown in the inset. The LOD measured as three times the signal of the blank is found to be about 1.7 μg/mL.

## 3. Experimental

### 3.1. Materials

Graphite fine powder (E.P.) was obtained from Tianheng Technology Co. Ltd. (Hong Kong SAR, China). Hydrazine hydrate of A.R grade (N_2_H_4_·H_2_O, 60 wt. %) was supplied by Oriental Chem. and Lab. Supplies Ltd. (Hong Kong SAR, China). β-cyclodextrin (C_42_H_70_O_35_, 96%) was purchased from Aladdin Chemistry Co. Ltd. (Nanqiao Town, Fengxian District, Shanghai, China). Rhodamine B (C_28_H_31_N_2_O_3_Cl, dye content ~95%) was obtained from Sigma-Adrich. All other chemicals were purchased from Oriental Chem. and Lab. Supplies Ltd. (Hong Kong SAR, China) and used as received.

### 3.2. Synthesis of GO

GO was synthesized by a modified Hummers method as described elsewhere [61]. Briefly, graphite fine powder (2.0 g) and NaNO_3_ (1.0 g) were placed in a flask (250 mL), followed by addition of concentrated H_2_SO_4_ (50 mL) in small portions under magnetic stirring and cooling by an ice bath. The mixture was kept stirring at 5 °C for 2 h, and then KMnO_4_ (7.3 g) was added portionwise under cooling and continuously stirring conditions to maintain the temperature of the suspension lower than 20 °C. Afterwards, the temperature was increased to 35 °C, and the reaction mixture was stirred continuously at this temperature for 30 min. DI water (90 mL) was then added gradually, and the mixture was stirred for another 15 min. Additional DI water (55 mL) was added, followed by slow addition of H_2_O_2_ (7 mL, 30%) for the purpose of reducing the residual permanganate to soluble manganese ions. Finally, the mixture was filtered and washed thoroughly with diluted HCl (5%, 150 mL), DI water (150 mL) and methanol (150 mL), and then vacuum-dried at 50 °C for 24 h.

### 3.3. Synthesis of CD-G

As-prepared GO powder (30.0 mg) and DI water (60.0 mL) were placed in a beaker, followed by sonication treatment for 1 h, which yielded a homogeneous yellow-brown suspension of graphene oxide (0.5 mg/mL). Separately, β-cyclodextrin (1.6 g) was added to a flask loaded with DI water (40.0 mL), and then magnetically stirred at 50 °C to dissolve the β-cyclodextrin. Afterwards, a portion of graphene oxide suspension (24 mL), ammonia (0.8 mL) and hydrazine hydrate (25.0 μL) were homogenized into the β-cyclodextrin solution, followed by a reaction at reflux under constant stirring at 80 °C for 3.5 h, leading to a black dispersion. The as-formed dispersion was charged into a dialysis bag for dialysis treatment against flowing DI water for 3 days, further washed repeatedly with DI water (50 mL) followed by ethanol (50 mL), vacuum-filtered using a nylon membrane with 0.22 um pore size, and finally dried under vacuum at 50 °C for 24 h. The resulting black powder was stored in a desiccator prior to use.

### 3.4. Fabrication of the Inclusion Complex of CD-G and RhB

The as-synthesized CD-G powder (26.5 mg) was homogenously dispersed into DI water (100 mL) by ultrasonication for 1 h. The obtained CD-G dispersion (0.265 mg/mL) was employed as the fluorescence nanoquencher. The fluorescence solution of RhB (50 nM) was prepared by dissolving the fluorescence dye RhB (0.012 mg) in DI water (500 mL). The CD-G dispersion (0.265 mg/mL) was mixed into the RhB solution (50 nM, 100 mL) in small portions by mild stirring each for 3 min to allow the sufficient interactions between RhB molecules and CD-G sheets. The gradual quenching process can allow monitoring of the RhB fluorescence quenching behavior by taking the digital images and recording the fluorescent spectra. The final inclusion complex of CD-G and RhB, with completely quenched fluorescence determined by the fluorescence spectroscopy, was used as the fluorescence switch-on probe for detection of a wide range of organic liquids.

### 3.5. Characterization

UV/Vis spectra were measured on a Lambda 18 UV/Vis spectrometer. FTIR spectra were recorded on a FTIR spectrometer (PerkinElmer System 2000) in KBr pellet mode. The powder XRD patterns were collected by a Bruker D8 Advance X-ray diffractometer (Bruker AXS, Karlsruhe, Germany). TGA tests were performed on a Mettler Toledo TGA/SDTA851 under N_2_ atmosphere at a heating rate of 10 °C/min. The surface morphologies were observed by a field emission scanning electron microscopy (FE-SEM, JEOL JSM-6335F). TEM images and SAED patterns were obtained by a Jeol JEM-2011 TEM facility at an acceleration voltage of 100 kV. The specimens for the TEM observation were prepared by dropping a diluted ethanol dispersion of the GO and graphene specimens onto a silicon wafer, followed by drying at room temperature. Fluorescence spectra were recorded on a Perkin-Elmer Luminescence spectrometer (LS50B), which was equipped with a motor-driven linear polarizer on the detection side, at room temperature under isotropic excitation.

## 4. Conclusions

An organic liquids-responsive graphene-based fluorescence probe has been fabricated in the present study by a simple, efficient and cost-effective method. To fabricate the probe, a one-pot chemical reduction and functionalization of GO with hydrazine in the presence of β-CD has been firstly conducted, which leads to the β-CD-modified and functionalized graphene. The β-CD has been demonstrated to be grafted on the graphene surfaces effectively and uniformly, as evidenced by various characterization techniques such as UV/Vis, FTIR, XRD TGA, SEM and TEM. The subsequent fabrication of the fluorescence probe has been achieved by inclusion of RhB molecules into CD-G using a facile and economical solution mixing method. The RhB fluorescence can be quenched controllably based on FRET from RhB to graphene through changing the mixing ratio of CD-G to RhB. A wide range of organic solvents including water-soluble and -insoluble ones has been tested using the fluorescence probe, which shows the highest sensitivity to THF, with the LOD of about 1.7 μg/mL. Some insight into the mechanism for explaining the different fluorescence responses of the probe to the examined targets has been provided. This work thereby shows a great significance for sensing and detection applications in a relatively unexplored field of detecting organic liquids using graphene-based platform, especially for the waste water containing THF contaminant, e.g., solvent leakage detection of tanks and piping systems. Based on the fluorescence spectroscopy, quantitative analysis of organic solvent concentration in water can also be realized using our developed graphene-based sensor. This work on the fabrication of the label-free graphene-based fluorescence probe by a simple, efficient and cost-effective strategy has opened up new avenues for development of other effective graphene-based probes for various sensing, monitoring and detection applications.
